# Supplementary material for: CircAgtpbp1 Acts as a Molecular Sponge of miR-543-5p to Regulate the Secretion of GH in Rat Pituitary Cells
Source: Animals (Basel). 2021 Feb 20;11(2):558. doi: 10.3390/ani11020558 (PMC7924184; doi:10.3390/ani11020558)
Supplement: Supplementary file 1 [file animals-11-00558-s001.pdf]

animals

MDPI

Supplementary Materials: CircAgtpbp1 Acts as a Molecular Sponge of miR-543-5p to Regulate the Secretion of GH in Rat Pituitary Cells

ZeWen Yu †, WenZhi Ren †, Tian Wang, WeiDi Zhang, ChangJiang Wang, HaoQi Wang, Fei Gao * and Bao Yuan *

Table S1. Primers and sequences used in this study.

	**Primers for qPCR**	
U6	RT	CGCTTCACGAATTTGCGTGTCAT
U6	F	GCTTCGGCAGCACATATACTAAAAT
	R	CGCTTCACGAATTTGCGTGTCAT
miR-543-5p	RT	CTCAACTGGTGTCGTGGAGTCGGCAATTCAGTT-GAGCGAAAAAC
	F	ACACTCCAGCTGGGAAGTTGCCCGCGTGT
	R	Universal Reverse Primer（CTCAAGTGTCGTGGAGTCGGCAA）
GADPH	F	GGAAACCCATCACCATCTTC
	R	GTGGTTCACACCCATCACAA
Gh1	F	CATGCCCTTGTCCAGTCTGT
	R	AATGTAGGCACGCTCGAACT
CircAgtpbp1	F	TGACCTCATTCTGAACTCTGACA
	R	TCTCGACTTCATTTTCAGCTTCT
Agtpbp1	F	TTACCATGCGAAGGGGCTAC
	R	GTCTTCCACAACATCGCAGG
	SiRNAs	
CircAgtpbp1 si-1		GCCAGTTTAACTACGTGGA
CircAgtpbp1 si-2		AGTTTAACTACGTGGACGA
CircAgtpbp1 si-3		TTAACTACGTGGACGACGT
	Probes for FISH	
Cy3-circAgtpbp1		RiboBio
	MiRNA sequences	
Mimic NC		RiboBio
miR-543-5p mimic		RiboBio
Inhibitor NC		RiboBio
MiR-543-5p inhibitor		RiboBio

File S1. The full sequence of circAgtpbp1.

The results of sequencing the full sequence of circAgtpbp1 was as follows:

TGGACGACGTGGTGGACGAGAGTGACGACAACGATGACATTGATTTAGAA
GCTGAAAATGAAGTCGAGAATGAAGATGACCTAGATCAAAGTTTTAAGAATGA
TGATATTGAAACAGATATTAATAAATTAAGACCCCAGCAAGTACCAGGACGAA
CAATAGAAGAACTAAAAATGTATGAGCACCTTTTCCCTGAGCTTGTTGATGATTT
TCAGGACTATGAATTAATCGCTAAAGAACCCAAACCTTTTGTGTTTGAGGGGAA
AGTTCGGGGCCCGATTGTAGTTCCCACAGCTGGAGAGGAAGTGCCTGGGAATCC
AGGTAACGTAAGGAAAGGAGCTGCAGTGAAGGAGAAAGCGAGTCCTAAAGGA
GAGGAAGTCAAGGAAGATGCCAAGGGCCATGACAAAACACCGCCGTGGCAGCT
GGGTGGCCAGAACAGAGCGGCCGCTTCAGCCCACAGCTCCAACAACGATCTTGT

GAAGGCCTTAGACCGAATCACACTGCAGAGTACCCCTTCACAAGTAGCCGCGG
GCTTGACTGCAGGAATGAGGAAGGACTACGGCCTCCCTCTCACTGTCCTCTCATG
CACGAAAGCGTGTCCTCACGTGGCTAAGTGCACAAGTGCCCTTTTCGAAGGGCG
GACAGTACATCTTGGTAAACTGTGTTGTACTGGAGTTGAAACGGAAGATGATGA
AGACTTTGAGTCCCACTCATCAGCAGAGCAGGTCTCCTCTGTTGAAGCCTCTGAT
GGACCACCAACACTGCATGACCCAGACCTCTACATCGAGATTGTGAAAAATACA
AAGTCTGTTCCCGAGTACTCAGAGGTGGCCTATCCTGATTATTTTGGACACATTC
CACCTCCCTTCAAAGAGCCTATTTTAGAAAGGCCTTATGGTGTACAAAGGACAA
AAATTGCCCAAGATATCGAGAGGCTGATACACCAGAATGATATCATTGACCGGG
TGGTGTATGACTTAGATAACCCTAACTATACCACTCCAGAAGAAGGAGATATTT
TGAAGTTTAACTCAAAATTTGAATCTGGGAATCTGCGCAAAGTAATTCAAATTA
GAAAAAGCGAGTATGACCTCATTCTGAACTCTGACATAAACAGTAACCATTACC
ATCAGTGGTTCTACTTTGAAGTCAGTGGGATGCGGCCGGGTGTGGCATACAGGTT
CAACATCATCAACTGTGAGAAGTCCAACAGCCAGTTTAACTACG

When the sequences was aligned in NCBI, 100% identity was observed. A total sequence of 1227 bp was identified, which showed 100% identity.

File S2. Construction of pmiR-circAgtpbp1-WT reporter plasmid and pmiR-circAgtpbp1-MUT reporter plasmid.

About 200 sequences upstream and 200 sequences downstream of circAgtpbp1 targeted binding to miR-543-5p was cloned between the XhoI and NotI sites in the pmirGLO plasmid, forming the pmiR-circAgtpbp1-WT plasmid. The target sequence AAAACAC was mutated into GGGGTGT, forming the pmiR- circAgtpbp1-MUT plasmid

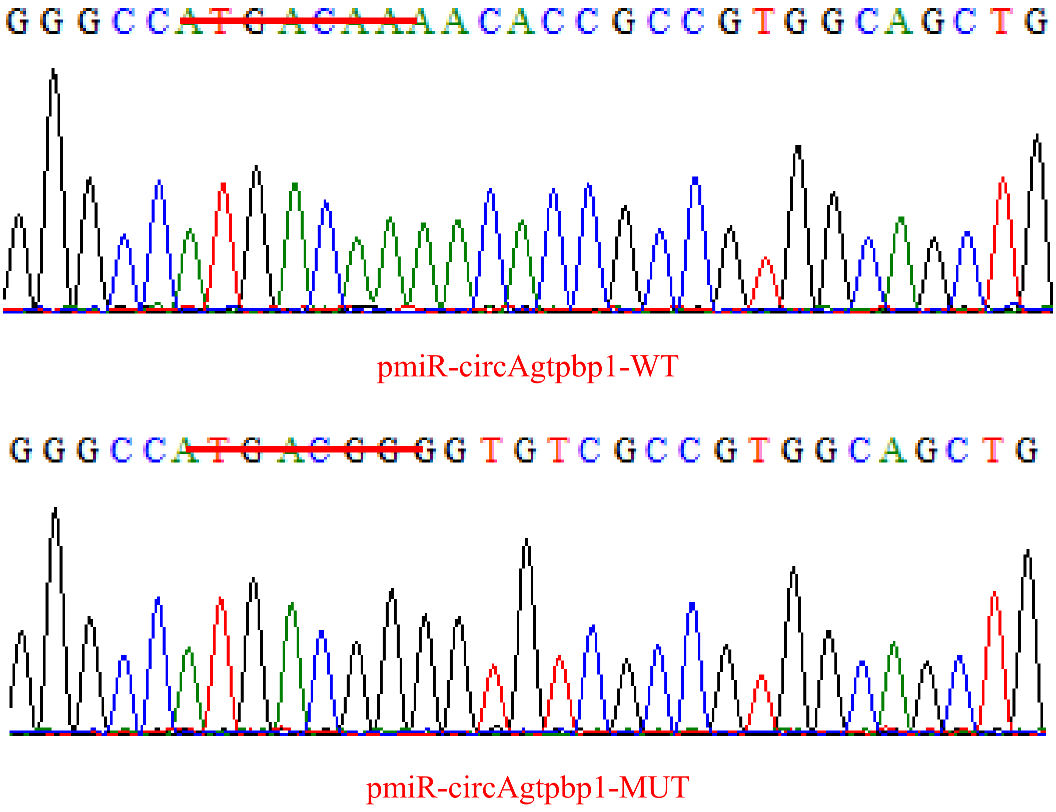

Figure S1. DNA sequence peak map. Sequence of the extracted plasmid; the target sequence AAAACAC was mutated into GGGGTGT.
